# Single-Dose Immunization With a Chimpanzee Adenovirus-Based Vaccine Induces Sustained and Protective Immunity Against SARS-CoV-2 Infection

**DOI:** 10.3389/fimmu.2021.697074

**Published:** 2021-06-28

**Authors:** Mingxi Li, Jingao Guo, Shuaiyao Lu, Runhong Zhou, Hongyang Shi, Xuanling Shi, Lin Cheng, Qingtai Liang, Hongqi Liu, Pui Wang, Nan Wang, Yifeng Wang, Lili Fu, Man Xing, Ruoke Wang, Bin Ju, Li Liu, Siu-Ying Lau, Wenxu Jia, Xin Tong, Lin Yuan, Yong Guo, Hai Qi, Qi Zhang, Zhen Huang, Honglin Chen, Zheng Zhang, Zhiwei Chen, Xiaozhong Peng, Dongming Zhou, Linqi Zhang

**Affiliations:** ^1^ NexVac Research Center, Comprehensive AIDS Research Center, Beijing Advanced Innovation Center for Structural Biology, School of Medicine, Tsinghua University, Beijing, China; ^2^ University of Chinese Academy of Sciences, Beijing, China; ^3^ Chinese Academy of Sciences, Shanghai, China; ^4^ National Kunming High-Level Biosafety Primate Research Center, Institute of Medical Biology, Chinese Academy of Medical Sciences and Peking Union Medical College, Kunming, China; ^5^ State Key Laboratory of Medical Molecular Biology, Department of Molecular Biology and Biochemistry, Institute of Basic Medical Sciences, Medical Primate Research Center, Neuroscience Center, Chinese Academy of Medical Sciences, School of Basic Medicine, Peking Union Medical College, Beijing, China; ^6^ AIDS Institute, Li Ka Shing Faculty of Medicine, The University of Hong Kong, Hong Kong, China; ^7^ State Key Laboratory for Emerging Infectious Diseases, Department of Microbiology, Li Ka Shing Faculty of Medicine, The University of Hong Kong, Hong Kong, China; ^8^ Institute for Hepatology, National Clinical Research Center for Infectious Disease, Shenzhen Third People’s Hospital, Shenzhen, China; ^9^ The Second Affiliated Hospital, School of Medicine, Southern University of Science and Technology, Shenzhen, China; ^10^ Department of Biomedical Engineering, School of Medicine, Tsinghua University, Beijing, China; ^11^ Tsinghua-Peking Center for Life Sciences, Tsinghua University, Beijing, China; ^12^ Laboratory of Dynamic Immunobiology, Institute for Immunology, Tsinghua University, Beijing, China; ^13^ Department of Basic Medical Sciences, School of Medicine, Tsinghua University, Beijing, China; ^14^ Department of Pathogen Biology, School of Basic Medical Sciences, Tianjin Medical University, Tianjin, China; ^15^ Teaching Center for Writing and Communication, Tsinghua University, Beijing, China; ^16^ Walvax Biotechnology Co., Ltd., Kunming, China; ^17^ School of Life Sciences, Tsinghua University, Beijing, China; ^18^ Beijing Key Laboratory for Immunological Research on Chronic Diseases, Tsinghua University, Beijing, China; ^19^ Beijing Frontier Research Center for Biological Structure, Tsinghua University, Beijing, China; ^20^ Shanghai Public Health Clinical Center, Fudan University, Shanghai, China

**Keywords:** SARS-CoV-2 vaccine, chimpanzee adenovirus vector, spike protein, single-dose immunization, protective immunity

## Abstract

The development of a safe and effective vaccine against SARS-CoV-2, the causative agent of pandemic coronavirus disease-2019 (COVID-19), is a global priority. Here, we aim to develop novel SARS-CoV-2 vaccines based on a derivative of less commonly used rare adenovirus serotype AdC68 vector. Three vaccine candidates were constructed expressing either the full-length spike (AdC68-19S) or receptor-binding domain (RBD) with two different signal sequences (AdC68-19RBD and AdC68-19RBDs). Single-dose intramuscular immunization induced robust and sustained binding and neutralizing antibody responses in BALB/c mice up to 40 weeks after immunization, with AdC68-19S being superior to AdC68-19RBD and AdC68-19RBDs. Importantly, immunization with AdC68-19S induced protective immunity against high-dose challenge with live SARS-CoV-2 in a golden Syrian hamster model of SARS-CoV-2 infection. Vaccinated animals demonstrated dramatic decreases in viral RNA copies and infectious virus in the lungs, as well as reduced lung pathology compared to the control animals. Similar protective effects were also found in rhesus macaques. Taken together, these results confirm that AdC68-19S can induce protective immune responses in experimental animals, meriting further development toward a human vaccine against SARS-CoV-2.

## Introduction

The rapid and global spread of the severe acute respiratory syndrome coronavirus 2 (SARS-CoV-2), the causative agent of coronavirus disease-2019 (COVID-19), calls for urgent development of safe, effective, and equitably accessible vaccines. Since the release of the genome sequence of SARS-CoV-2 in early January of 2020 (1), scientists and industrial partners around the world have been working tirelessly to develop various vaccines based on traditional and innovative platforms. For example, the mRNA vaccines developed by Pfizer/BioNtech and Moderna have recently been approved for emergency use by several regulatory agencies (2). The entire pipeline relies on innovative technologies for both antigen design and gene expression, which offer unprecedented speed and flexibility (3–5). However, the long-term safety, efficacy, and durability of protection require further characterization. The adenovirus-based vaccines, developed by CanSino (Ad5) (6), Gamaleya (Ad5 and Ad26) (7), AstraZeneca/Oxford University (ChAdOX1) (8), and Johnson & Johnson (Ad26) (9), have also incorporated novel features for antigen and vector optimization and have recently been approved for emergency use by several regulatory agencies. However, those based on human adenovirus serotype 5 (Ad5) may compromise their efficacy due to preexisting immunity to the vector. The rare adenovirus serotype vectors originated from human or chimpanzee may circumvent such limitation (10). Nevertheless, given their prior experience in the adenovirus vector-based vaccines against Ebola, Middle East Respiratory Syndrome Coronavirus (MERS-CoV) and human immunodeficiency virus type I (HIV-1) (11–13), this strategy is likely to be superior in large-scale manufacturing, distribution, and administration of vaccines, which may practically translate into larger accessibility and greater protection at the population level. Furthermore, killed vaccines developed by Sinopharm and Sinovac also demonstrated impressive safety and efficacy profiles in human populations around the world. This classic form of vaccine has a proven record of success in inducing protective immunity in humans against various viral pathogens, although potential risks exist when manufacturing and handling industrial amounts of live infectious particles (14). Additional vaccine platforms, based on DNA, recombinant proteins, or novel viral vectors, are also being developed (15–17).

The reported vaccine candidates incorporate either the full-length spike (S) protein of SARS-CoV-2 or only its receptor-binding domain (RBD) as the immunogen, since the RBD of S plays critical roles in mediating viral entry (18–20) and inducing a protective antibody response in infected individuals as well as experimental animals (21–25). Like other coronaviruses, the S protein of SARS-CoV-2 consists of a globular S1 domain, an N-terminal region, a membrane-proximal S2 domain, and a transmembrane domain (18). The RBD, which is located within the S1 domain, determines the host range and cellular tropism, while the S2 domain mediates membrane fusion (18). SARS-CoV-2 infects airway epithelial cells *via* an interaction of the RBD with the cellular receptor angiotensin-converting enzyme 2 (ACE2) (26). At the initial outbreak of SARS-CoV-2, we and others resolved the crystal structure of the SARS-CoV-2 RBD bound to ACE2, which revealed that the overall ACE2-binding mode is nearly identical to that of the SARS-CoV RBD (20, 26–28). This suggests that agents capable of disrupting this binding interaction could serve as candidates to block the entry of SARS-CoV-2 into target cells. Indeed, both polyclonal and monoclonal antibodies directed against the RBD and the S protein in general have been shown to inhibit SARS-CoV-2 infection and provide protection against infection or diseases in experimental animals (21, 25, 29–31), reinforcing the scientific rationale to incorporate S or the RBD as vaccine immunogens.

The current work aims to develop a novel SARS-CoV-2 vaccine based on a derivative of less commonly used rare adenovirus serotype AdC68 vector. Apart from low pre-existing immunity in humans, this vector was demonstrated to induce strong and long-lasting immunity against various viral antigens in experimental animals (32–34). For example, the AdC68 vector was used by our group to develop a vaccine candidate expressing the full-length MERS-CoV S glycoprotein. Single-dose intranasal administration of this vaccine completely protected human DPP4 knock-in mice from lethal MERS-CoV challenge. Passive transfer of immune sera also conferred a survival advantage in lethal challenge mouse models (32). This is in great contrast to the immunity induced by other candidate vaccines, for which immunogenicity is short-lived, and multiple rounds of immunization are required to induce detectable levels of neutralizing antibodies or to confer protection against viral challenge (3, 10). Furthermore, recombinant vaccines based on AdC68 and other rare serotype chimpanzee adenoviral vectors, such as ChAd63 and ChAd3, have recently been engineered to express various antigens, with some demonstrating impressive safety and immunogenicity profiles in clinical studies (13, 35–37). These unique features and our prior experience in handling the chimpanzee adenovirus vectors provided a critical foundation and rationale for the development of a SARS-CoV-2 vaccine based on AdC68.

Here, we report on the construction and characterization of three vaccine candidates using AdC68 expressing either the full-length spike (AdC68-19S) or receptor-binding domain (RBD) with two different signal sequences (AdC68-19RBD and AdC68-19RBDs). We showed that single-dose intramuscular immunization with the three vaccines induced robust and sustained neutralizing antibody responses in BALB/c mice up to 40 weeks after immunization, with AdC68-19S being superior to AdC68-19RBD and AdC68-19RBDs. Importantly, immunization with AdC68-19S was able to induce strong and protective immunity against a live-virus challenge in both golden Syrian hamsters and rhesus macaques, supporting the further development of AdC68-19S into a clinical vaccine against SARS-CoV-2 infection in humans.

## Materials and Methods

### Construction, Rescue, Amplification and Purification of AdC68-Based Vaccines

The AdC68 is an E1- and E3-deleted replication-deficient adenoviral vector (38). It also has a partial deletion in E4, which was replaced by the corresponding E4 region of the human adenovirus 5 (Ad5). The codon-optimized gene encoding the spike (S) protein from SARS-CoV-2 (Wuhan-Hu-1, GenBank: MN908947.3) was synthesized by Tsingke Biological Technology, China. The coding sequence of the RBD region, which spans the residues Arg319–Phe541 of the S protein, was obtained by PCR from the codon-optimized spike gene. The full-length S protein, RBD with the original signal peptide from the S protein, and RBD with the secretory signal peptide from the mouse Ig heavy chain were inserted into the E1-deleted region of the AdC68 vector by isothermal assembly to obtain the vectors pAdC68-19S, pAdC68-19RBD, and pAdC68-19RBDs, respectively. Primers used for PCR are listed in **Supplementary Materials**. Empty AdC68 vector with no insertion in the E1 deletion region was employed as the negative control vaccine. The vectors pAdC68-19S, pAdC68-19RBD, and pAdC68-19RBDs were linearized and transfected into HEK293 cells (ATCC, CRL-1573-ATC) to rescue AdC68-19S, AdC68-19RBD, and AdC68-19RBDs. Theses recombinant adenoviruses as well as empty control AdC68 were propagated and purified by CsCl gradient ultracentrifugation before quantification by spectrophotometry (38).

### Detection of S and RBD Protein Expression

HEK293T cells cultured in 6-well plates were infected with AdC68-19S, AdC68-19RBD, AdC68-19RBDs, and empty AdC68 at doses of 10^10^, 10^9^, and 10^8^ vp per well. After 24 h, the cells were harvested and lysed with 100 μl of buffer containing a protease inhibitor cocktail. The cell lysates were subjected to SDS-PAGE, followed by western blotting with a primary anti-SARS-CoV-2 S antibody (Sino Biological, China) and detected using a horseradish peroxidase (HRP)-conjugated secondary anti-rabbit IgG (Promega, USA). Beta-actin was used as a loading control. In the flow cytometry assay, S and RBD expression was detected by surface and intracellular staining, respectively. Two human monoclonal antibodies (P2C-1F11 and P2B-2F6) (21) specific for the RBD isolated from SARS-CoV-2 infected individuals were incubated with infected cells at a final concentration of 10 μg/ml at 4°C for 30 min. After extensive washing, the cells were further incubated with anti-human IgG-PE (BioLegend, USA) at a 1:50 dilution and analyzed using a BD Calibur FACS instrument (BD, USA). The VRC01 antibody specific for human immunodeficiency virus type I (HIV-1) was used as a negative control.

### Mouse Immunization and Sample Collection

A total of 50 female BALB/c mice aged 6–8 weeks were randomly divided into 10 groups (n = 5 in each group) and immunized with a single dose comprising 10^10^, 10^9^, or 10^8^ vp of AdC68-19S, AdC68-19RBD, and AdC68-19RBDs, or AdC68 *via* intramuscular (IM) route in a volume of 100 μl. Serum samples were collected every 2 weeks until 8 weeks following the immunization, heat-inactivated at 56°C for 30 min, and stored at −80°C before analysis for SARS-CoV-2 specific antibodies. T cell responses were measured in the spleens of 10 additional female BALB/c mice one week after immunization. To further study the durability of the elicited antibody response, 10 additional female BALB/c mice aged 6–8 weeks were intramuscularly immunized with 10^10^ vp of AdC68-19S and followed up to 40 weeks. Blood samples were collected every 2 weeks, heat-inactivated at 56°C for 30min, and stored at −80°C before analysis for SARS-CoV-2-specific binding and neutralizing antibodies.

### Golden Syrian Hamster Immunization and Challenge With Live SARS-CoV-2

The entire procedure was performed as described previously (39). Six to eight-week-old-male and female hamsters were obtained from the Chinese University of Hong Kong Laboratory Animal Service Centre through the HKU Laboratory Animal Unit and kept in Biosafety Level-2 (BSL-2) housing. The hamsters (n = 4 per group) were immunized with AdC68-19S or saline as control *via* the IM route in a volume of 100 μl. Blood samples were collected at week 2 and week 4 post administration for antibody detection. Six weeks after immunization, animals were transferred to the BSL-3 animal facility and intranasally challenged with 10^5^ PFU of live SARS-CoV-2 (HKU-13 strain, GenBank accession no: MT835140) in a volume of 100 μl under intraperitoneal ketamine (200 mg/kg) and xylazine (10 mg/kg) anesthesia. The weights of the hamsters were monitored daily and the animals were sacrificed at day 4 post-challenge. Lung tissues were collected for viral loads determination and immediately fixed in 10% PBS-buffered formalin for histopathological and immunohistochemical observation. The viral loads were determined using a quantitative SARS-CoV-2 nucleocapsid/*β*-actin reverse transcription-polymerase chain reaction assay as described before (39). Plaque forming unit (PFU) of lung tissues was also measured. Lung homogenates were diluted in an appropriate dilution, then added into pre-seeded Vero-E6. After 3 days, the number of plaques in the 12 well plate was observed, and the PFU was calculated. For immunostaining, lung tissue sections were incubated with rabbit anti-SARS-CoV nucleocapsid (NP) protein antibody (1:4,000, Sino Biological, Beijing, China) at 4°C, overnight. This was followed by FITC-conjugated donkey anti-rabbit IgG secondary antibody (Jackson ImmunoResearch, PA, USA) for 30 min at room temperature. The mean fluorescence intensity (MFI) of NP positive cells was then examined and counted under a fluorescence microscope. Paraffin-embedded tissues were cut into 4 μm sections and stained with hematoxylin and eosin (H&E) for histopathological observation.

### Rhesus Macaque Immunization and Challenge With Live SARS-CoV-2

Four adult male rhesus macaques aged between 5 and 9 years were intramuscularly vaccinated with 10^11^ vp of AdC68-19S or empty AdC68 in a volume of 200 μl. Peripheral blood samples were collected every 2 weeks for antibody. The vaccinated animals were intratracheally challenged with 10^6^ PFU of live SARS-CoV-2 (Wuhan-HU-1 strain, GenBank accession no: NC_045512.2) in a volume of 1 ml 8 weeks after intramuscular immunization. Nasal swabs were collected on days 0, 1, 2, 3, 5, and 7 after the viral challenge. The animals were euthanized on day 7 after the challenge, and the lungs were collected for viral load analysis and histopathological examination. Viral gRNA and sgRNA in the lung tissues and nasal swabs were measured by droplet digital PCR (40), using a COVID-19 digital PCR detection kit (TargetingOne, China). The kit allows the detection of the ORF1ab gene, N gene, and a positive reference gene. The limit of detection is 100 copies/ml. The same detection kit with different primers and probes to target the E gene was used for the detection of sgRNA (41). For histopathological analysis, lung tissues were collected and fixed in 10% neutral buffered formalin, embedded in paraffin, and sectioned (5 μm) for standard hematoxylin and eosin staining.

### Binding Antibodies Measured by ELISA

For the immunized mice, the serum samples were serially diluted and added to 96-well plates pre-coated with recombinant SARS-CoV-2 S trimer or RBD produced in HEK 293F cells (100 ng/well). After incubation at 37°C for 1 h, the plates were washed three times with phosphate-buffered saline containing 0.1% Tween^®^ 20 (PBST) and incubated with a secondary HRP-conjugated antibody against mouse IgG (1:4,000, Promega, USA), IgG1, IgG2a, IgG2b, or IgA (1:40,000; Abcam, UK) at 37°C for 1 h. The samples were further washed three times with PBST before the substrate TMB (3′3′,5′,5′-tetramethyl benzidine) was added, and the reaction was stopped by adding 1 M H_2_SO_4_. Absorbance at 450 nm was measured using an ELISA plate reader. The ED50 value was calculated based on binding curves drawn in Prism 8.0 software (GraphPad Inc., USA). For immunized golden Syrian hamsters, SARS-CoV-2 RBD (50 ng/well; Sino Biological, Beijing, China) specific binding antibody titers were detected with HRP-conjugated goat anti-hamster IgG (Invitrogen, Carlsbad, CA, USA). The ED50 value was calculated based on binding curves drawn in Prism 8.0 software (GraphPad Inc., USA). For immunized rhesus macaques, the IgG response specific to the SARS-CoV-2 S trimer or RBD was measured using an HRP-conjugated anti-monkey IgG (1:6,000; Southern Biotech, USA). The endpoint antibody titer of the macaque sera was defined as the optical value three times above that of the native serum.

### Neutralizing Antibodies Measured by Pseudovirus and Live SARS-CoV-2

Neutralizing titers of the immune sera were determined using SARS-CoV-2 pseudovirus and live virus neutralization assays as previously reported (21). The pseudovirus was generated by co-transfection of HEK293T cells with the HIV backbone expressing firefly luciferase (pNL43R-E-luciferase) and pcDNA3.1 (Invitrogen, USA) encoding S proteins from the wild type S protein Wuhan-Hu-1 (GenBank: MN908947.3). The variant B.1.1.7 (GISAID: EPI_ISL_601443) was constructed with mutations 69–70del, 144del, N501Y, A570D, D614G, P681H, T716I, S982A, and D1118H. The variant B.1.351 (GISAID: EPI_ISL_700450) was constructed with mutations L18F, D80A, D215G, 242-244del, S305T, K417N, E484K, N501Y, D614G, and A701V. The variant P.1 (GISAID: EPI_ISL_792681) was constructed with mutations L18F, T20N, P26S, D138Y, R190S, K417T, E484K, N501Y, D614G, H655Y, T1027I, and V1176F. After 48 h, the cell supernatant containing the pseudovirus was collected, measured, and stored at −80°C until further use. Serum samples were serially diluted three-fold in 96-well cell culture plates before SARS-CoV-2 pseudovirus was added and incubated at 37°C for 1 h. Approximately 1.5 × 10^4^ Huh7 cells were then added to the serum–pseudovirus mixture and incubated at 37°C for an additional 60 h. The ID50 values were calculated based on the relative light units (Bright-Glo Luciferase Assay Vector System, Promega, USA) using Prism 8.0 (GraphPad Software Inc., USA). For the live virus assay, we used live SARS-CoV-2 (Beta/Shenzhen/SZTH-003/2020, EPI_ISL_406594 at GISAID) initially isolated from an infected patient in China, and the focus reduction neutralization was performed in a certified BSL3 facility at Shenzhen Third People’s Hospital, China. Briefly, serial dilutions of sera were mixed with SARS-CoV-2 and incubated for 1 h at 37°C. The mixtures were then transferred to 96-well plates seeded with Vero E6 cells and incubated for 1 h at 37°C. After changing the medium, the plates were incubated at 37°C for an additional 24 h. The cells were then fixed, permeabilized, and incubated with cross-reactive rabbit anti-SARS-CoV-N IgG (Sino Biological, Inc., China) for 1 h at room temperature before adding an HRP-conjugated goat anti-rabbit IgG antibody (Jackson ImmunoResearch, USA). The reactions were developed using KPL TrueBlue peroxidase substrate (Seracare Life Sciences Inc., USA). The number of SARS-CoV-2 foci was quantified using an EliSpot reader (Cellular Technology Ltd. USA).

### AdC68 Neutralization Assay

Serum samples were three-fold serially diluted in 96-well cell culture plates then mixed with 1,500 TCID50 of green fluorescent protein expressed AdC68-GFP viruses. Then the mixtures were incubated at 37°C for 1 h. After incubation, 1.5 × 10^4^ of HEK293A cells was added to each well cocultured for 24 h. The green fluorescent protein levels were examined by Opera Phenix (PerkinElmer) to determine the vector-specific neutralizing antibodies. Half-maximal inhibitory dilutions (ID50) of AdC68 were defined as the dilution required to reduce green fluorescent protein-expressing cells by 50% compared to wells with virus alone.

### Assessment of T-Cell Responses in Mice

Cellular immune responses in the vaccinated mice were assessed using the IFN-*γ* pre-coated ELISPOT kit (MabTech, Sweden) according to the manufacturer’s protocol. Splenocytes from immunized mice were stimulated with a peptide pool covering the SARS-CoV-2 S protein (GenScript, USA) at a concentration of 2 μg/ml of each peptide. Phorbol myristate acetate/ionomycin was used as a positive control, and RPMI 1640 medium as a negative control. After incubation at 37°C for 28 h, the plates were washed extensively before a biotinylated anti-mouse IFN-*γ* antibody was added to each well and incubated further for 2 h at room temperature. The substrate AEC was added, and the spots in each well were read using the automated ELISPOT reader (AID, USA). The number of spot-forming units (SFUs) per 1,000,000 cells was calculated. For the intracellular cytokine staining, approximately 1,000,000 mouse splenocytes were stimulated with the same SARS-CoV-2 S peptide pool as above (2 μg/ml of each peptide) and brefeldin A (GolgiPlug; BD, USA) for 6 h at 37°C in 5% CO_2_. Following two washes with PBS, the splenocytes were permeabilized and stained with the fluorescently conjugated antibodies CD4-FITC (GK1.5; BioLegend, USA), CD8-PE/Cyanine7 (53-6.7; BD), CD19-APC/Cyanine7 (1D3; BD), INF*γ-*BV421 (XMG1.2; BD), TNFα-APC (MP6-XT22; BioLegend), and IL2-PE (JES6-5H4, BioLegend). Dead cells were stained using the Zombie Yellow Fixable Viability Kit (BioLegend, USA). The data were collected using a Cytek Aurora FACS instrument (Cytek, USA) and analyzed using FlowJo software.

### Ethics Statement

All experiments were carried out in strict compliance with the Guide for the Care and Use of Laboratory Animals of the People’s Republic of China and approved by the Committee on the Ethics of Animal Experiments of Tsinghua University, Chinese Academy of Medical Sciences, and University of Hong Kong. Mouse immunization and characterization were conducted in the animal facility of Tsinghua University. Golden Syrian hamster experiments involving live SARS-CoV-2 were conducted in the ABSL-3 facility at the University of Hong Kong. Rhesus macaque experiments involving live SARS-CoV-2 were performed in the ABSL-4 facility of the Kunming National High-level Biosafety Primate Research Center, Yunnan, China, and approved by the institutional biosafety committee.

### Statistical Analysis

Prism 8.0 software (GraphPad, USA) was used for statistical analysis and data plotting. Unless specified otherwise, the data are presented as means ± SEM. Analysis of unpaired Mann–Whitney test (two-tailed) was used to determine the statistical significance of differences among different groups (*P < 0.05; **P < 0.01; ***P < 0.001; ****P < 0.0001; ns, no significance).

## Results

### Construction and Characterization of Recombinant AdC68 Vaccines Expressing the Spike and RBD of SARS-CoV-2

**Figure 1:**
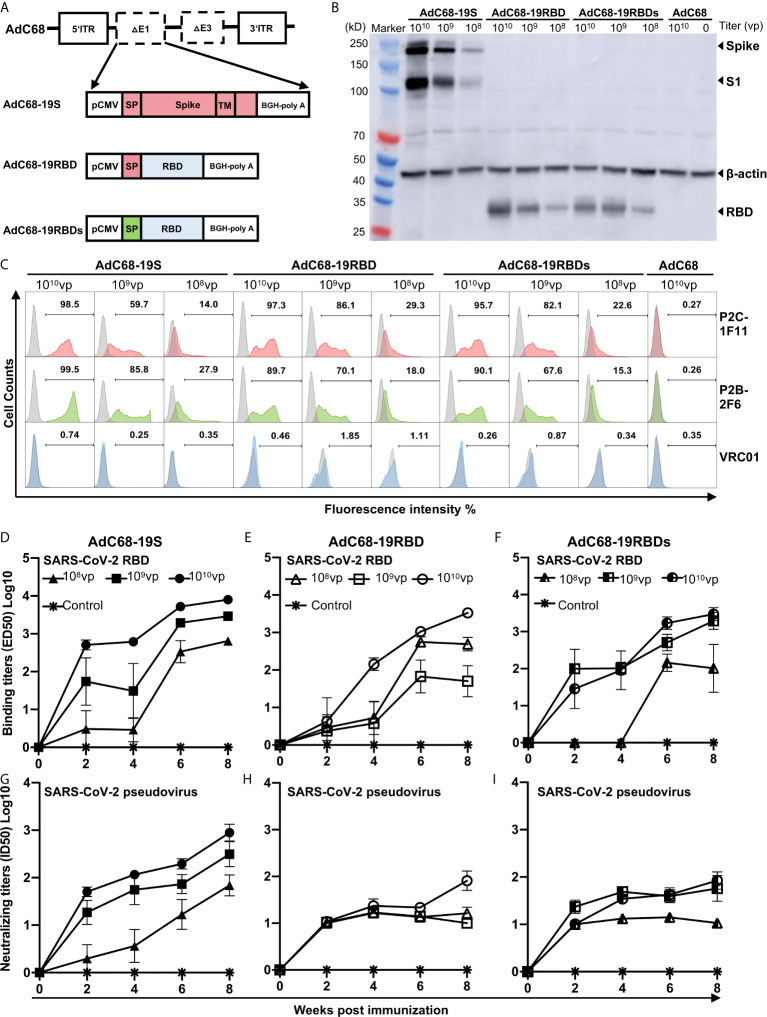
Generation and characterization of recombinant AdC68 expressing the full-length spike protein or RBD of SARS-CoV-2. **(A)** Schematic diagram of the recombinant AdC68 expressing the full-length S protein with its original signal peptide (AdC68-19S), RBD with the original signal peptide from the S protein (AdC68-19RBD), or RBD with the secretory signal peptide from mouse IgG (AdC68-19RBDs). The coding sequence of the S protein or RBD was inserted into the E1 region of the AdC68 vector under the control of the CMV promoter and terminated using a bovine growth hormone (BGH) polyadenylation signal sequence. **(B)** Western blot analysis of S and RBD expression in HEK293T cells infected with the recombinant AdC68 (10^8^, 10^9^ and 10^10^ vp). **(C)** Flow cytometry analysis of S and RBD expression using the SARS-CoV-2-specific mAbs P2C-1F11 and P2B-2F6. HEK293T cell lysates and intact HEK293T cells infected with the empty AdC68 vector (10^10^ vp) were used as negative controls. VRC01 was used as a negative control antibody. BALB/c mice (n = 5 per group) were immunized by three vaccines respectively. The empty AdC68 was used as a control vaccine. Serum binding activity of total IgG to SARS-CoV-2 RBD **(D–F)** and neutralizing activity against pseudotyped SARS-CoV-2 **(G–I)** over an 8-week period after single-dose immunization with AdC68-19S, AdC68-19RBD, or AdC68-19RBDs. For each recombinant AdC68 construct, three different doses (10^10^, 10^9^, and 10^8^ vp) were administrated through IM route, which is indicated by different symbols. Data points corresponding to animals in the empty AdC68 vector control groups (10^10^ vp) are shown with asterisk. All data are presented as means ± SEM.

We generated three recombinant AdC68 vaccines, expressing either the full-length S protein with the original signal peptide (AdC68-19S), the RBD with the original signal peptide of the S protein (AdC68-19RBD), or the RBD with a secretory signal peptide (AdC68-19RBDs) (**Figure 1A**). The coding sequences of S or RBD were inserted into the E1 region of the AdC68 vector under the control of the CMV promoter and terminated with a bovine growth hormone (BGH) polyadenylation signal sequence. HEK293T cells infected with AdC68-19S, AdC68-19RBD, AdC68-19RBDs showed the desired expression of S or RBD according to both western blot (**Figure 1B**) and flow cytometry analysis (**Figure 1C**). Dose-dependent expression of S, S1, and RBD with the expected molecular weight was detected in all infected cells, while none was found in cells infected with the empty vector AdC68 as expected (**Figure 1B**). Surface expression of S and intracellular expression of the RBD were further confirmed by staining with the RBD-specific mAbs P2C-1F11 and P2B-2F, initially isolated from SARS-CoV-2 infected individuals (**Figure 1C**). As both mAbs recognize conformational epitopes on the RBD, the positive signals indicated proper expression and presentation of RBD epitopes by the infected cells. Here again, dose-dependent expression was also found by both western blot and flow cytometry analysis, while no signal was detected using the negative control antibody VRC01 (**Figure 1C**).

To study the immunogenicity of the recombinant vaccines, we inoculated 10 groups of six-week-old BALB/c mice (n = 5 per group) with AdC68-19S, AdC68-19RBD, or AdC68-19RBDs. Each regimen consisted of three single doses (10^10^, 10^9^, or 10^8^ vp) administered through the IM route. The negative control animals only received a dose of 10^10^ vp of the AdC68 empty vector. Serum samples were collected every 2 weeks for 8 weeks, and their binding and neutralizing activities against SARS-CoV-2 pseudovirus were analyzed. As shown in **Figures 1D–I**, all three recombinant vaccines were able to induce strong and durable antibody responses after a single immunization. Binding affinity for the RBD and neutralizing activity against pseudovirus became detectable 2 weeks after immunization and continued to rise throughout the 8-week period, particularly for the AdC68-19S group. Furthermore, dose-dependent responses were found for all vaccine candidates. By week 8 after immunization, the AdC68-19S animals had an overall average binding ED50 of 8585.4 in 10^10^vp group while those AdC68-19RBD, and AdC68-19RBDs animals had respective values of 3527.2 and 3948.5. For neutralizing activity, the AdC68-19S elicited an average ID50 of 1253.9, which were approximately 10-fold higher than in the animals vaccinated with AdC68-19RBD (172.5) and AdC68-19RBDs (115.0). No detectable binding and neutralizing activities were found among the negative control animals. These results indicate that AdC68-19S, AdC68-19RBD, and AdC68-19RBDs are all immunogenic, but AdC68-19S was superior in inducing both binding and neutralizing antibody responses in BALB/c mice.

### AdC68-19S Induced Potent and Durable Immune Responses Against SARS-CoV-2 in BALB/c Mice

**Figure 2:**
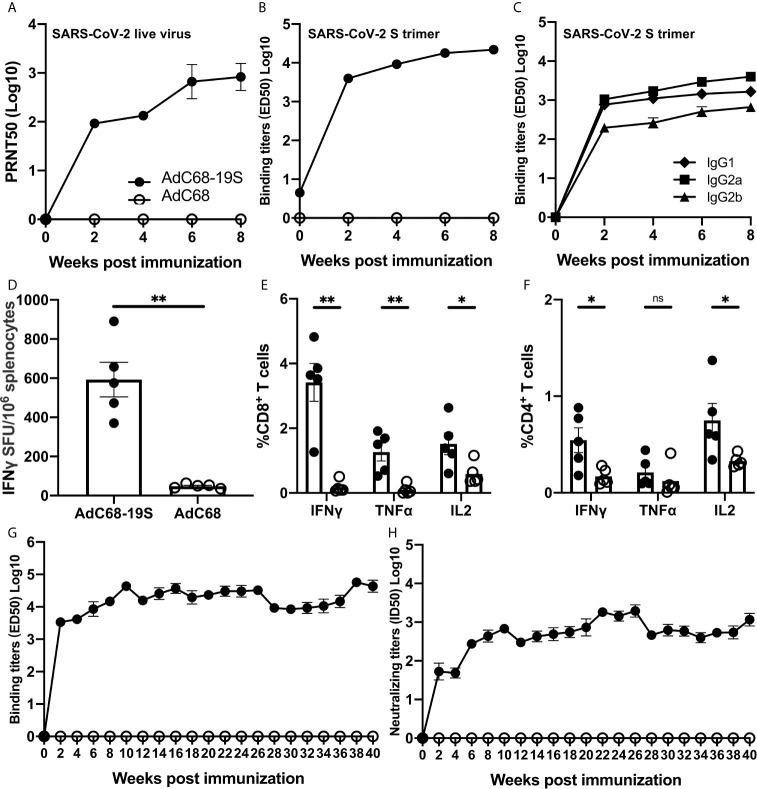
AdC68-19S induces strong and durable immune responses in BALB/c mice. Animals immunized with 10^10^ vp of AdC68-19S were characterized in greater detail. **(A)** Neutralizing activity of the immunized serum against live SARS-CoV-2 over an 8-week period after immunization. **(B)** Binding activity of the immune serum total IgG to SARS-CoV-2 S trimer. **(C)** Binding activity of immune serum IgG subtypes IgG1, IgG2a, and IgG2b to SARS-CoV-2 S trimer. **(D)** ELISPOT analysis of IFN-*γ*-positive splenocytes and FACS analysis of intracellular IFN-*γ*, TNF-α, and IL-2 production in **(E)** CD8^+^ and **(F)** CD4^+^ splenic T cells one week after immunization. The durability of the antibody response was analyzed using **(G)** ELISA of total IgG with the SARS-CoV-2 S trimer and **(H)** pseudovirus neutralization up to 40 weeks after single-dose immunization. Data corresponding to animals in the AdC68-19S group are shown in solid circle, and those in the AdC68 group in hollow circle. All data are presented as the means ± SEM. Analysis of unpaired Mann–Whitney test was used to determine the statistical significance of differences among different groups (*P < 0.05; **P < 0.01; ns, no significance).

We next conducted a more in-depth analysis of the antibody and T-cell responses in animals immunized with the highest dose of AdC68-19S (10^10^ vp) (**Figure 2**). Consistent with what was detected in the pseudovirus neutralization assay, the neutralizing activity against live SARS-CoV-2 became detectable 2 weeks after immunization and continued to rise throughout the 8-week period. The neutralizing antibody titer (PRNT ID50) reached to 957.3 by week 8. No neutralizing activity was detected in animals vaccinated with the empty AdC68 vector as expected (**Figure 2A**). The total serum IgG binding to the S trimer reached an average ED50 of 23058.0 by week 8 after immunization (**Figure 2B**), of which, IgG2a appeared to be dominant, followed by IgG1 and IgG2b throughout the entire study period (**Figure 2C**). Splenocytes collected 1 week after immunization were subjected to interferon (IFN)-*γ* ELISPOT and intracellular cytokine staining (ICS) analysis. In the vaccine group, 593 spot-forming cells (SFU) per 10^6^ splenocytes were detected after simulation with an overlapping peptide pool of the S protein, whereas those in the negative control group were as low as 48 SFU per 10^6^ splenocytes) (**Figure 2D**). The ICS results also showed the same trend in the number of CD8^+^ and CD4^+^ T cells producing IFN-*γ*, tumor necrosis factor (TNF)-α, and interleukin (IL)-2 (**Figures 2E, F**). These results indicate that a single immunization with AdC68-19S induces strong antibody and cellular immune responses by IM route.

To further study how durable antibody response after a single immunization, we intramuscularly inoculated 10 six-week-old BALB/c mice with either 10^10^ vp of AdC68-19S (five animals) or 10^10^ vp of the empty AdC68 vector as a negative control (five animals). Blood samples were collected every 2 weeks from the time of immunization until 40 weeks thereafter. As shown in **Figure 2G**, the titer of binding antibodies against the S trimer increased dramatically two weeks after immunization, continued to increase and remained at high levels in the ensuing period. Furthermore, the neutralizing antibody response, measured using the pseudovirus neutralization assay, also demonstrated a similar trend of increase and persistence after a single immunization. These results indicate that a single immunization with AdC68-19S through IM route can induce strong and durable antibody response, a unique and advantageous feature compared to other reported strategies.

### AdC68-19S Induced Protective Immunity in Golden Syrian Hamsters of SARS-CoV-2 Infection

**Figure 3:**
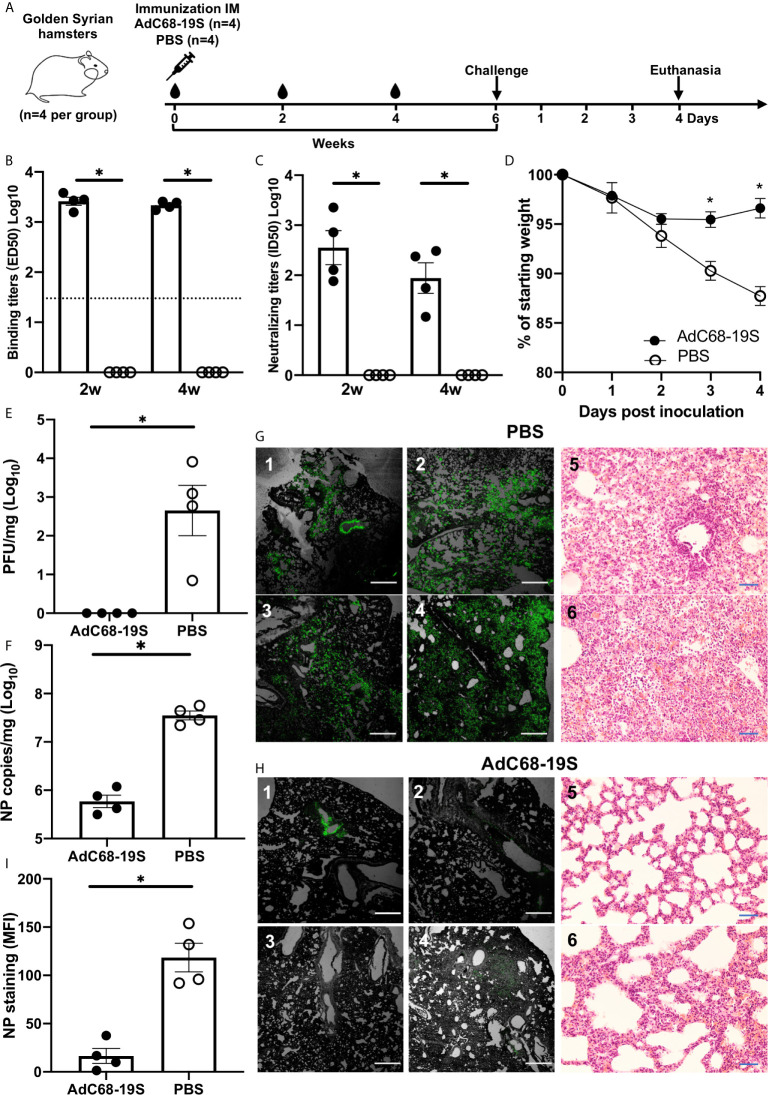
AdC68-19S elicits a protective immune response against SARS-CoV-2 infection in golden Syrian hamsters. **(A)** Timeline for vaccination, challenge with live SARS-CoV-2 and virological, immunological, and pathological characterization. The hamsters were immunized with AdC68-19S (n = 4) or saline as control (n = 4) *via* the IM route. **(B)** The binding ability of total IgG and neutralizing ability of immune serum to SARS-CoV-2 RBD on week 2 and week 4 after immunization. **(C)** The neutralizing ability of the serum samples against SARS-CoV-2 pseudovirus. **(D)** Weight loss in SARS-CoV-2 challenged hamsters. Lung viral load measured by **(E)** plaque forming unit (PFU) and **(F)** qPCR. Immunostaining of NP antigen in lung tissue sections detected by a fluorescent monoclonal antibody against SARS-CoV-2 nucleocapsid protein and hematoxylin and eosin staining (20×) **(G**, **H)**. The scale bar was 500 μm for immunostaining and 100 μm for HE staining. **(I)** The actual values of mean florescence intensity (MFI) measured. Data corresponding to animals in the AdC68-19S group are shown in solid circle, and those in the PBS group in hollow circle. Dotted lines reflect assay limit of detection. All data are presented as the means ± SEM. Analysis of unpaired Mann–Whitney test was used (*P < 0.05).

To evaluate the protective potential of AdC68-19S, we intramuscularly immunized a total of eight hamsters with either 10^10^ vp of AdC68-19S (n = 4) or saline as control (n = 4) and challenged with 10^5^ plaque-forming units (PFU) of SARS-CoV-2 (HKU-13 strain, GenBank accession no: MT835140) 6 weeks post immunization (**Figure 3A**). Serum binding and neutralizing antibody were evaluated before challenge, while body weight changes, lung infectious viral titer, lung viral RNA copies, lung immunohistochemical staining for viral nucleocapsid protein (NP), and lung histopathological outcome were evaluated 4 days after challenge. As shown in **Figures 3B, C**, AdC68-19S elicited a strong binding and neutralizing antibody response at 2 and 4 weeks after immunization. Immunization protected the animals from body weight loss (**Figure 3D**) and significantly reduced number of infectious virus (**Figure 3E**) and viral RNA copies (**Figure 3F**) in the lungs compared to the control animals. Immunohistochemistry analysis detected abundant NP widely distributed along the alveolar epithelia cells in the control group but only sporadically in the AdC68-19S immunized animals (**Figures 3G–I**). Histopathological analysis also consists of the result of immunohistochemical staining (**Figures 3G, H**). The alveolar structure of AdC68-S vaccinated animals showed slight inflammatory cell infiltration; most of them maintained the normal structure. Conversely, the severe pathological changes of alveolar structure such as alveoli collapse and infiltration were observed in the control group. Collectively, these results show that AdC68-19S can elicit protective immunity in the golden Syrian hamster model of SARS-CoV-2 infection.

### AdC68-19S Elicited Protective Immune Responses Against SARS-CoV-2 in Rhesus Macaques

**Figure 4:**
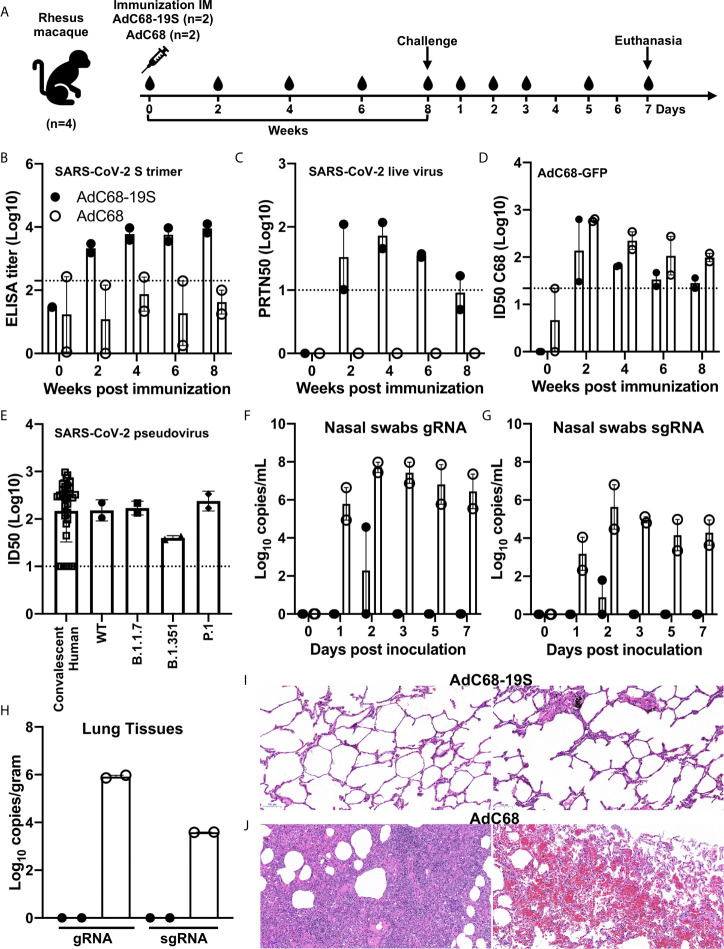
AdC68-19S induces protective immune responses in rhesus macaques. **(A)** Timeline for vaccination, challenge, euthanasia, and virological and immunological characterization. Four animals were intramuscularly immunized with 10^11^ vp of AdC68-19S (n = 2) or 10^11^ vp of the empty AdC68 vector (n = 2). Eight weeks post-immunization, the animals were challenged with 10^6^ PFU of live SARS-CoV-2. Blood samples were collected every 2 weeks before viral challenge. Blood samples and nasal swabs were collected on days 0, 1, 2, 3, 5, and 7 after the challenge. All animals were euthanized on day 7 after the challenge to quantify the viral load in the lung tissue and conduct histopathological examinations. Binding activity of immune serum total IgG to the SARS-CoV-2 S trimer **(B)**. Neutralizing activity of immune serum against live SARS-CoV-2 **(C)**. **(D)** AdC68-vector neutralizing antibodies in serums were assessed by AdC68-GFP neutralization assays. **(E)** Neutralizing activity of immune sera from convalescent patients and rhesus macaques 6 weeks post IM immunization in pseudovirus neutralization against the WT strain and variants. Viral gRNA and sgRNA copies in nasal swabs **(F**, **G)** and in lung tissue samples from six lobes **(H)** were quantified by droplet digital PCR (TargetingOne, China). **(I, J)** Histopathological comparison of tissues from animals vaccinated with AdC68-S and AdC68. Representative tissue sections in standard hematoxylin and eosin staining are shown (10×). The scale bar is 100 μm. Data corresponding to animals in the AdC68-19S group are shown in solid circle, and those in the control group in hollow circle. Dotted lines reflect assay limit of detection. All data are presented as the means ± SEM.

We went further to confirm the protective potential of AdC68-19S in rhesus macaque model of SARS-CoV-2 infection, although only four animals were available due to the severe shortage during the pandemic. Two animals each received single intramuscular immunization of either 10^11^ vp of AdC68-19S or AdC68 empty vector control, challenged with 10^6^ PFU of live SARS-CoV-2 by intratracheal inoculation 8 weeks post vaccination, and euthanized on day 7 after the challenge (**Figure 4A**). As shown in **Figures 4B, C**, single immunization with AdC68-19S induced both binding antibody to S trimer and neutralizing antibody against live SARS-CoV-2 throughout the 8 week period before challenge. While binding antibody became relatively stable 4 weeks after immunization, neutralizing titer peaked around the same period but waned in the ensuing time. We also assessed the vector-specific neutralizing antibodies (**Figure 4D**). All AdC68-19S- or empty AdC68-vector-vaccinated animals developed low levels of AdC68 vector-specific neutralizing antibodies, as reported previously (34, 42). The ID_50_ titer against the AdC68 vector peaked 2 weeks post-immunization and declined quickly over time. However, the neutralizing titer appeared to be higher in empty AdC68-vaccinated than in AdC68-19S-vaccinated animals. In terms of neutralizing antibody against SARS-CoV-2 pseudovirus, AdC68-19S-vaccinated macaques induced similar levels compared to convalescent serum from 35 SARS-CoV-2 patients in Beijing. The mean titers measured in ID50 were comparable between the two groups (152.3 *vs.* 170.5) (**Figure 4E**). To determine whether immune sera from AdC68-19S-vaccinated macaques could neutralize the SARS-CoV-2 variants of concern, we assessed the sera against four pseudoviruses carrying either the wild type Wuhan-Hu-1 strain, B.1.1.7 variant (GISAID: EPI_ISL_601443), B.1.351 variant (GISAID: EPI_ISL_700450), and P.1 variant (GISAID: EPI_ISL_792681). Compared with the ID50 against WT pseudovirus, the immune sera from the rhesus macaques at week 6 demonstrated about 3.9-fold decline against B.1.351, while it remained relatively unchanged or slightly increased against B.1.1.7 and P1, respectively (**Figure 4E**). Furthermore, immunized animals showed remarkable levels of protection against SARS-CoV-2 challenge compared to the controls. AdC68-19S-vaccinated animals only experienced transient levels of gRNA or sgRNA in the nasal swabs, while the control animals showed persistent high levels throughout the experiments (**Figures 4F, G**). No detectable levels of gRNA or sgRNA were found in the lung tissues of AdC68-19S-vaccinated macaques, while in the control group the viral load reached as high as 8.36 × 10^5^ and 3.89 × 10^3^ copies per gram (**Figure 4H**). Finally, histopathological analysis on the lung sections collected on day 7 after the viral challenge showed that the AdC68-19S-vaccinated macaques maintained a normal lung structure with mild infiltration of interstitial lymphocytes and macrophages recruited to the alveolar space (**Figure 4I**). By contrast, the control animals showed severe interstitial pneumonia in all lobes, as evidenced by the infiltration of monocytes and lymphocytes in most alveoli, as well as edema in a proportion of alveoli (**Figure 4J**). These results indicate that AdC68-19S was able to elicit protective immune responses against SARS-CoV-2 infection in rhesus macaques.

## Discussion

Adenovirus-based vaccine strategies represent a major component in our efforts against SARS-CoV-2 infection. Among those approved for emergency use by the regulatory authorities around the world, at least four vaccines used adenoviruses although the genotype and serotype varied among different vectors (6–9). In this study, we explored another rare chimpanzee adenovirus serotype AdC68 as vector to construct and evaluate vaccine against SARS-CoV-2 infection. The AdC68 vector appears to be phylogenetically and serologically distinctive from the current adenovirus vectors used for SARS-CoV-2 vaccines such as the abovementioned Ad5, Ad26, and ChAdOX1 (43, 44), providing an additional and alternative candidate to this class of vaccine used either alone or in combination. Specifically, we show that a single intramuscular immunization of AdC68-19S elicited a robust and sustained neutralizing antibody response in BALB/c mice up to 40 weeks after immunization. It also induces protective immunity in Syrian hamsters from weight loss and significantly reduces viral burdens in the lungs and respiratory tract and lung pathology. Similar protective effect was also found in rhesus macaque model of SARS-CoV-2 infection. These results indicate that AdC68-19S can induce protective immune responses in experimental animals and merit further development of AdC68-19S toward a human vaccine against SARS-CoV-2 either singly or as prime-boost combinations with heterologous vectors.

A couple of unique aspects of our study can be highlighted here. First and foremost, the AdC68 vector has desirable properties such as a large capacity for foreign genes and low pre-existing immunity in humans (10, 45, 46). This is clearly superior to many adenoviral vectors that are widely used in vaccine development, such as human adenovirus 5 (Ad5), whose seroprevalence in the normal human population reaches as high as 75–80% (45–49). By contrast, the seroprevalence of AdC68 is only 0–15%, and even when it is positive, the serum antibody titer is generally low (46, 48, 49). This unique feature allows enhanced immunogenicity at low doses of antigens expressed by AdC68, thus reducing the likelihood of adverse effects. Previous studies have proved that the preexisting immunity to Ad2,4,5,7,12 does not affect the immune responses induced by chimpanzee adenoviral vector including AdC68 (44, 50). Furthermore, the AdC68 vector appears to be serologically and phylogenetically distinct from Ad5, Ad26, and ChAdOx1 (43, 44). In fact, the parental AdC68 vector has been engineered to express antigens from a wide range of pathogens such as Ebola virus, HIV-1, and influenza A virus, and demonstrated impressive safety and immunogenicity profiles in preclinical studies (33, 34, 51–53). In addition, vaccines based on other serotypes of chimpanzee adenovirus showed similar profiles of low pre-existing immunity. In particular, the ChAdOx1 vaccine expressing the full-length of SARS-CoV-2 S protein (AZD1222), developed by AstraZeneca/Oxford University, has showed promising safety and efficacy against SARS-CoV-2 infection despite some trial participants only receiving a partial dose (8, 54, 55). The same adenovirus vector expressing the full-length S protein of MERS-CoV was also evaluated in a phase I human trial and demonstrated good safety and tolerability (11). ChAd3 vectors expressing Ebola Zaire glycoprotein (ChAd3-EBO-Z) elicited strong immune responses in clinical trial participants (56, 57). These results highlight the favorable safety, tolerability, and immunogenicity profiles of chimpanzee adenovirus vector-based vaccines in humans, holding great promise for vaccine development. Although, there were some safety concerns associated with rAd-based SARS-CoV-2 vaccines, the mechanism of thrombosis remains unclear. The rAd vectors including ChAdOx1 and Ad26 have been tested in hundreds and thousands of individuals and demonstrated impressive safety profile before application to SARS-CoV-2 vaccine. The thrombosis found in small proportion of SARS-CoV-2 vaccinees was a big surprise to many investigators, suggesting AEs could only be identified when a large number of individuals were studied. Several studies have demonstrated that severe cases of frequently manifested abnormal platelet activity, suggesting the spike protein might somehow enhance thrombosis in the later stage of disease (58–60). Further investigation is needed to figure out the potential relationship between blood clot and adenovirus vaccine. In our ongoing clinical trial (ChiCTR2100046612), we will closely monitor vaccinated volunteers and conduct relevant studies to ensure safety. The dose of AdC68-19S in the clinical trial was 5 × 10^10^ vp, which is comparable with that of ChAdOX1 nCoV-19 (8, 54, 55). In the study of macaques, due to the limited number of animals, we only tested the immunogenicity at the high dose of 10^11^ vp. From the results of mice, we found that both 10^10^ vp and 10^9^ vp can induce strong and durable antibody responses. Therefore, it is feasible to lower the dose in the consideration of safety in clinical trials. The second unique aspect of this study is the exceptional durability and strength of the protective immunity elicited by AdC68-19S in experimental animals. In BALB/c mice, a single intramuscular immunization of AdC68-19S elicited robust and sustained immune responses lasting for up to 40 weeks post immunization. Such high and persistent levels of antibodies are superior to many vaccines for which immunogenicity is short-lived, and multiple rounds of immunization are required to induce a detectable and protective immune response (3). While the underlying mechanism is currently unclear, it is possible that the broad host-cell tropism of AdC68 and the expression of membrane-anchored instead of soluble S protein by the recombinant AdC68-19S played critical roles. Taken together, these unique features enhance AdC68-19S capacity to induce potent and protective immune responses against SARS-CoV-2 in experimental animals and provide scientific rational for further developing AdC68-19S toward a clinical vaccine against SARS-CoV-2 infection in humans.

## Data Availability Statement

The original contributions presented in the study are included in the article/**Supplementary Material**. Further inquiries can be directed to the corresponding authors.

## Ethics Statement

The animal study was reviewed and approved by the Committee on the Ethics of Animal Experiments of Tsinghua University, Chinese Academy of Medical Sciences, and University of Hong Kong.

## Author Contributions

DZ and LZ conceived, designed, and supervised the entire study. ML, JG, HS, MX, and WJ constructed, produced, and purified the vaccines and carried out all immunogenicity evaluations in mice. SL, HL, and XP performed the immunization and protection experiment in rhesus macaques. RZ, PW, LL, SL, HC, and ZC performed the immunization and protection experiments in golden Syrian hamsters. QL, LF, QZ, and XS assisted the mouse immunization and evaluation experiments. LC, BJ, and ZZ carried out the live virus neutralization assays. NW and YG carried out the droplet digital PCR. YW and HQ assisted the intracellular cytokine staining assays. RW made the construction of S variants. ZH, XT, and LY are employees of Walvax Biotechnology Co., Ltd. ML, GJ, DZ, and LZ wrote the manuscript. All other authors reviewed and edited the manuscript. All authors contributed to the article and approved the submitted version.

## Funding

This work was chiefly supported by funds from the National Key Plan for Scientific Research and Development of China (2020YFC0848800, 2020YFC0844200, 2018ZX10731101-002, 2017ZX10201101, 2016YFD0500307), the Beijing Advanced Innovation Center for Structural Biology, National Natural Science Foundation of China (31870922, 32070926, 81530065 and 91442127), and the Science and Technology Innovation Committee of Shenzhen Municipality (202002073000002). It was also supported by Tsinghua University Initiative Scientific Research Program (20201080053), Beijing Municipal Science and Technology Commission (171100000517 and Z201100005420019), and Tsinghua University Spring Breeze Fund (2020Z99CFG004), as well as Tencent Foundation, Shuidi Foundation, and TH Capital. ZC and HC are partially supported by the InnoHK CVVT program.

## Conflict of Interest

The authors declare that LZ, DZ, ML, and XS are co-inventors on pending patent applications related to the AdC68-19S, AdC68-19RBD, and AdC68-19RBDs vaccine candidates. ZH, XT, and LY are employees of Walvax Biotechnology Co., Ltd.

The remaining authors declare that the research was conducted in the absence of any commercial or financial relationships that could be construed as a potential conflict of interest.
